# *Vibrio vulnificus* mutation rate: an *in vitro* approach

**DOI:** 10.3389/fmicb.2023.1223293

**Published:** 2023-08-09

**Authors:** Francisco Jose Roig Molina, Carmen Amaro González, Alejandro Alcaine Otín, Jesús Carro Fernández

**Affiliations:** ^1^Computing for Medical and Biological Applications Group, Facultad de Ciencias de la Salud, Universidad San Jorge, Zaragoza, Spain; ^2^Departamento de Microbiología y Ecología, Universidad de Valencia, Valencia, Spain; ^3^Estructura de Investigación Interdisciplinar en Biotecnología y Biomedicina BIOTECMED, University of Valencia, Valencia, Spain

**Keywords:** mutation rate, number of generations, *Vibrio vulnificus*, lineages, evolution

## Abstract

*Vibrio vulnificus* is a multi-host pathogenic species currently subdivided into five phylogenetic lineages (L) plus one pathovar with the ability to infect fish due to a transmissible virulence plasmid. This plasmid (or a fragment of it) has been transmitted between lineages within the species, contributing to the evolution of *V. vulnificus*. This study aimed to provide an experimental approximation to the *V. vulnificus* mutation rate by determining spontaneous mutation rates from bacterial cultures of representants of the different lineages by whole-genome sequencing. To this purpose, synonymous SNP differences, i.e., spontaneous mutation not subjected to the evolutive forces, between initial and final culture after serial growth were evaluated and used for mutation rate calculation.

## Introduction

*Vibrio vulnificus* is an emerging zoonotic pathogen that inhabits brackish water ecosystems from temperate and subtropical areas. In recent years, the pathogen has expanded its geographical distribution to coastal areas near the Arctic Circle due to global warming (Baker-Austin et al., 2013, 2017). *V. vulnificus* survives in water, either associated with the mucous surfaces of algae and aquatic animals or as a free-living bacterium that can be concentrated by filtering organisms such as oysters, which are its main reservoir (Oliver, 2015; Baker-Austin and Oliver, 2020).

The diseases caused by this species are globally known as vibriosis. Fish vibriosis is a hemorrhagic septicemia that occurs in farms as epizootics or outbreaks of high mortality (Amaro et al., 2015). *V. vulnificus* infects fish by contact, colonizes the gills, enters the blood, and causes death by septicemia in <24 h (Marco-Noales et al., 2001). Human vibriosis can be acquired by either ingestion of raw seafood or contact of wounds with seawater or carrier fish (Oliver, 2015; Baker-Austin and Oliver, 2020). In the first case, the pathogen colonizes the intestine and causes gastroenteritis and/or primary septicemia. In the second case, the pathogen crosses the skin barrier causing local but severe necrosis and/or secondary septicemia. The main factor that predisposes to death by sepsis is a high level of serum iron because of multiple pathologies (e.g., hemochromatosis, diabetes, cirrhosis, and viral hepatitis) (Oliver, 2015; Baker-Austin and Oliver, 2020).

*V. vulnificus* was defined as a bacterial species in 1976 (Farmer, 1979) and was later split into three biotypes (Bts) based on a few biochemical and serological differences as well as host range (Amaro and Biosca, 1996; Biosca and Amaro, 1996; Biosca et al., 1997; Bisharat et al., 1999, 2005). According to this classification, Bt1 encompassed environmental and clinical strains isolated worldwide, Bt3 included clinical strains isolated from Israel, and Bt2 was formed by strains isolated from diseased fish (Linkous and Oliver, 1999; Raul et al., 2004; Jones and Oliver, 2009; Haenen et al., 2014; Heng et al., 2017). However, as more strains were isolated, more exceptions were found to the original biotype description, which showed that a new intraspecies classification was needed. A phylogenomic study based on the single nucleotide polymorphism (SNP) present in the core genome of 80 strains demonstrated that the species was subdivided into five lineages and a pathovar (pv. *piscis*) with the specific ability to cause disease in fish due to a transmissible virulence plasmid (Roig et al., 2018). The lineages with more isolates were L1 and L2, with L1 including most human isolates from infections acquired by the oral route (formerly classified as biotype 1) and L2 including most isolates from infections acquired by contact (formerly classified as biotype 1) plus all the isolates from diseased fish (pathovar *piscis*, formerly classified as Bt2) (https://www.frontiersin.org/files/Articles/319988/fmicb-08-02613-HTML-r1/image_m/fmicb-08-02613-g002.jpg). The remaining lineages also grouped environmental and clinical strains from specific geographical areas. This study also suggested that the virulence plasmid had been acquired independently several times, probably in the fish farming environment, and suggested that horizontal gene transfer events had contributed to the evolution of the species.

For evolution to take place, genetic variability is required and this can come from mutations or can be acquired from an external source through horizontal gene transfer (HGT). The balance between these two sources of variability can be drastically different between bacterial species. Some species, usually known as monomorphic species (Achtman, 2008), have low mutation rates (MRs) and rarely, if ever, exchange genetic material with other strains or species. This has not prevented them from becoming highly successful pathogens such as *Mycobacterium tuberculosis*. For others, recombination and horizontal gene transfer (HGT) are the main sources of variation. For example, Sánchez-Busó et al. (2015) estimated that ~98% of the SNPs incorporated in an outbreak-related lineage of *Legionella pneumophila* originated from recombination with other strains of the same species. In the case of *V. vulnificus*, we know that the acquisition of the virulence plasmid has been very important for the emergence of new lineages linked to fish farms (Amaro and Carmona-Salido, 2023).

The MR of a species can be very useful to predict the divergence time between strains or variants of the same species. To calculate MRs and divergence time, it is required to know the number of generations per year, the synonymous SNP positions, and the total number of synonymous sites between each pair of strains (Foster et al., 2009; Galloway-Peña et al., 2012). For these three parameters, there are no reliable data for *V. vulnificus*, although there are some data for specific genes with a very high MR (Broza et al., 2007). For comparative purposes, it has been estimated that *Escherichia coli* has ~100–300 generations/year (Ochman et al., 1999), *Bacillus anthracis* has 43 generations per year (van Ert et al., 2007), and *Vibrio parahaemolyticus* has 100 generations per year (García et al., 2012).

This study aimed to estimate the MR for both chromosomes of *V. vulnificus* in strains representative of three main phylogenetic lineages by using an “omic” approach through the identification of synonymous SNPs (sSNPs) present in the same strain after 30 days of serial 24 h cultures. Our estimate of the MR can be used to predict the divergence time between strains or variants, thus providing a temporal scale for relevant evolutionary and epidemiological events in *V. vulnificus*.

## Materials and methods

### Bacterial isolates, culture conditions, DNA extraction, and sequencing

The strains (Table 1) were routinely grown in tryptone soy broth or agar plus 5 g/l NaCl (TSB-1 or TSA-1, Pronadisa, Spain) at 28°C for 24 h. The strains were maintained both as lyophilized stocks and as frozen stocks at −80°C in marine broth (Difco) plus 20% (v/v) glycerol.

**Table 1:** Origin, year of isolation, biotype, serovar, virulence-related typing, and genome accession number of *V. vulnificus* strains used in this study.

**Strain**	**Source**		**Country**	**Year of isolation**	**Biotype/serovar or clade^a^**	**vvpdh^b^**	**vcg^b^**	**Lineage^c^**	**Sequence accession**	
									***T*** = **0**	***T*** = **30**
94385	Clinical	Human wound	Spain	2001	1	+	E	L4	SAMN11477453	SAMN11477454
CECT4604	Environmental	Diseased eel	Spain	1990	2/E	+	E	L2	SAMN11477455	SAMN11477456
162	Clinical	Human blood	Israel	1997	3/O	+	E	L3	SAMN11477451	SAMN11477452

^a^O-antigen serovar was determined for Bt2 and 3 isolates according to Biosca et al. (1997): Clade A and B were described by Danin-Poleg et al. (2015) and Efimov et al. (2015), respectively. NT, non-typable.

^b^vvpdh (V. vulnificus potentially dangerous for humans); pilF polymorphism associated with human virulence (Roig et al., 2010). vcg, virulence correlated gene; E, environmental type; C, clinical type.

^c^Lineages are defined by Roig et al. (2018). Strain CECT4604 belongs to pv. piscis.

### Serial cultures

Tubes containing 10 ml of fresh TSB-1 were inoculated with a single colony from TSA1-plates and were incubated at 28°C for 24 h. A volume of 100 μl from each grown tube was inoculated in a new tube containing 10 ml of the same medium and this was incubated again at 28°C for 24 h. The procedure was repeated daily for 1 month. To control the bacterial growth in each tube, the number of bacteria was estimated by drop plate (Herigstad et al., 2001) at times 0, previously to each new inoculation. Plates were incubated for 24 h at 28°C. The number of UFC was expressed in UFC/ml. This calculus shows the information referent to the number of daily inoculum and the growth rate of the day to check the proper evolution of the experiment.

### Number of generations

The number of generations was calculated using the expression (Maier, 2009) from the equation:


$$\begin{array}{l} Number\;of\;generations=log_{2}\left(\frac{cells\;at\;end\;of\;incubation}{cells\;at\;beginning\;of\;incubation}\right) \end{array}$$

where the initial number of bacteria is the initial inoculum (number of UFC) and the total number of bacteria is the accumulative number of bacteria obtained from the drop-plating counts.

### Sequencing, filtering reads, and adapter removal

DNA was extracted using GenElute™ Bacterial Genomic DNA (Sigma, Spain). Samples with a DNA concentration of 10–15 ng/μl were used for sequencing using the Illumina Genome Analyzer Technology GAII flow cell in the Genome Analysis Center in Norwich (UK). To this end, unique index-tagged libraries for each sample (up to 96 strains) were created using TruSeq DNA sample preparation for subsequent cluster generation (Illumina cBot), and up to 12 separate libraries were sequenced in each of eight channels in Illumina Genome Analyzer GAII cells with 100 base paired-end reads. The index-tag sequence information was used for downstream processing to assign reads to the individual samples (Harris et al., 2010). The quality of the FASTQ files was checked using FastQC (http://www.bioinformatics.babraham.ac.uk/projects/fastqc/). After the quality analysis, based on the results obtained, the adapters were removed using the program Cutadapt (Martin, 2011); after this step, the preprocessing of the data was carried out using Prinseq (Schmieder and Edwards, 2011). The preprocessing was carried out by filtering terminal indetermination in both ends (N), and sequences with mean quality (Phred quality score) lower than 26 or with more than 5% of indetermination or size smaller than 150 bp were removed. Finally, bases, in both ends, with quality lower than 28 were trimmed. A filtering quality of the reads was performed based on quality scores and the presence of Ns in the sequence. Finally, unpaired reads were removed from the preprocessed fastq files using a custom script. Raw data can be accessed as an SRA project with the accession number PRJNA534103.

### Variant calling

The preprocessed reads were mapped using the Bowtie2 software (Langmead and Salzberg, 2012), as previously described (Roig et al., 2018) using the corresponding genes of the strain YJ016 (BA000037.2—BA000038.2) as reference. As a reference to plasmids, selected based on previously described plasmid distribution in the species (Roig and Amaro, 2009), the plasmid of the strains YJ016 and CECT4602 (AP005352.1, AM293859, and AM293860) was used. The variant calling was performed using the pipeline GATK with Picard and Samtools (Li et al., 2009; McKenna et al., 2010; Pirooznia et al., 2014) using the default options of each tool.

### Mutation rate and divergence time calculation

The sSNPs were selected because we assume that they are neutral or nearly neutral in terms of selection and therefore allow for relatively unbiased estimation of SNP accumulation (Hershberg and Petrov, 2010). The previously described expression (Galloway-Peña et al., 2012).


$$\begin{array}{l} \;MR=\\ \frac{\;number\;of\;sSNPs}{\left(num.\;of\;possible\;sSNP\times num.\;of\;generations\;per\;year\times 2\right)}was\;used. \end{array}$$

Based on this expression, MR units are expressed as the number of mutations per base pair per generation (MBY).

Synonymous SNP sites were calculated by elucidating the sSNP sites for each codon within the core genome genes (Roig et al., 2018). The number of potential sSNP positions is listed in Table 2. The calculation was performed by analyzing all codons of the reference sequence, and for each codon, the positions where synonymous and non-synonymous changes can be produced were analyzed.

**Table 2:** Reference size and number of potential Syn sites.

**Compartment**		**94385**			**CECT4604**			**162**		
		**Num genes**	**Size**	**Possible Syn sites**	**Num genes**	**Size**	**Possible Syn sites**	**Num genes**	**Size**	**Possible Syn sites**
Core	ChroI	1,996	1,976,570	1,343,140	1,996	1,976,570	1,343,140	1,996	1,976,570	1,343,140
	ChroII	871	940,364	634,414	871	940,364	634,414	871	940,364	634,414
	Total	2,867	2,916,934	1,977,554	2,867	2,916,934	1,977,554	2,867	2,916,934	1,977,554
	Bt1 Conjugative Plasmid^*^	75	48,508	32,780	-	-	-	-	-	-
	Bt2 Conjugative Plasmid^*^	-	-	-	69	56,628	38,208	-	-	-
	Bt2 Virulence Plasmid^*^	-	-	-	67	66,946	44,628	-	-	-
Accessory	ChroI	1,388	1,035,716	692,018	1,680	1,609,024	1,076,734	1,770	1,742,460	1,177,911
	ChroII	841	731,734	491,216	1,402	1,355,566	906,425	709	696,624	468,617
	Total	2,229	1,767,450	1,183,234	3,082	2,964,590	1,983,159	2,479	2,439,084	1,646,528
	Bt1 Conjugative Plasmid^*^	-	-	-	-	-	-	-	-	-
	Bt2 Conjugative Plasmid^*^	-	-	-	-	-	-	-	-	-
	Bt2 Virulence Plasmid^*^	-	-	-	-	-	-	-	-	-

^*^Present in the core of the strains with those plasmids.

Finally, the VCF files obtained for each sampling time were compared to obtain the sSNPs. Those positions in which the change, synonymous or non-synonymous, occurred between sampling times, regardless of their base present in the reference, were considered as a change.

The time required for sequence differentiation between strains can be estimated from the number of SNPs accumulated during a known time of change between the ancestral and derived strains (Foster et al., 2009; Galloway-Peña et al., 2012; Sandberg et al., 2014). The following equation was used to obtain an approximate age of divergence for each pairwise comparison:


$$\begin{array}{l} \;Divergence\;time=\\ \frac{\;number\;of\;sSNPs}{\left(num\;of\;possible\;sSNP\times MR\times num.\;of\;generations\;per\;year\times \;2\right)}. \end{array}$$

## Results

### Number of generations

The final average number of generations calculated using the previously expressed formula turned out to be 215 (213 ± 2 for 94385, 214 ± 1 for 162, and 219 ± 4 for CECT4604) in 30 days of continuous culture experiment between the initial inoculum and the final culture. In the absence of real data for *V. vulnificus*, as previously mentioned, this number of generations will be considered the number of generations for 1 year of environmental growth.

### Characteristics of the genomes

Two different strains representative of lineages 2 and 3 plus one additional strain representative of one of the three geographically specific lineages (L4) were used in the study (Table 1). A total of 2,867 chromosomal genes conformed the core genome of the three strains, defined as the set of genes with a length of 80% and a DNA identity higher than 70% with respect to the homologous gene in the reference genome (Roig et al., 2018). The accessory genome contained 2,229 genes in strain 94385, 3,082 genes in strain CECT4604, and 2,479 genes in strain 162. The distribution of genes, total size, and synonymous sites in the three strains for the different chromosomes and plasmids are shown in Table 2.

The accessory genome contained 2,229 genes in strain 94385, 3,082 genes in strain CECT4604, and 2,479 genes in strain 162. From these accessory genes, 58 genes corresponded to a conjugative plasmid present in strain 94385, and 67 and 69 genes to virulence and conjugative plasmids, respectively, both present in strain CECT4604. The distribution of genes, total size, and synonymous sites in the three strains for the different chromosomes and plasmids are shown in Table 2.

### Mutation rate

The obtained average number of generations in 30 days was 215. This value corresponds to a generation time of 3.35 h under lab growth conditions.

MRs were estimated separately for the chromosomes and plasmids in the three studied strains (Tables 3–7). The average MR estimate for the core genome was 7.291E-08; for the accessory genome, the average MR was 1.370E-07; and for the pan genome, the average MR was 8.643E-08 mutations per base pair per generation. The MR estimated for each isolate was different, for CECT4604 and 162 strains, the MR was higher for the accessory chromosome II than for chromosome I. In contrast, strain 94385 showed a higher MR in the accessory genome of chromosome I than in the accessory genome of chromosome II; these changes also were reflected in a higher MR for chromosome I than in chromosome II for the pan genome (Tables 4, 5).

**Table 3:** Number of SNPs and MRs in core genome.

**Type**	**162**		**94385**		**CECT4604**	
	**Num sSNP**	**Mut. rate**	**Num sSNP**	**Mut. rate**	**Num sSNP**	**Mut. rate**
Number of total changes	140		95		201	
Number of synonymous changes	46	5.4095E-08	38	4.4688E-08	102	1.1995E-07
Number of non-synonymous changes	94		57		99	
Number of total changes in chromosome I	85		56		115	
Number of synonymous changes in chromosome I	28	4.8481E-08	22	3.8092E-08	57	9.8693E-08
Number of non-synonymous changes in chromosome I	57		34		58	
Number of total changes in chromosome II	55		39		86	
Number of synonymous changes in chromosome II	18	6.5983E-08	16	5.8651E-08	45	1.6496E-07
Number of non-synonymous changes in chromosome II	37		23		41	
Number of total changes in conjugative plasmid			1		7	
Number of synonymous changes in conjugative plasmid			0	0	0	0
Number of non-synonymous changes in conjugative plasmid			1		7	
Number of total changes in virulence plasmid					12	
Number of synonymous changes in virulence plasmid					1	5.211E-08
Number of non-synonymous changes in virulence plasmid					11	

**Table 4:** Number of SNPs and MRs in accessory genome (mutations per base pair per generation).

**Type**	**162**		**94385**		**CECT4604**	

	**Num sSNP**	**Mut. rate**	**Num sSNP**	**Mut. rate**	**Num sSNP**	**Mut. rate**
Number of total changes	65		439		98	
Number of synonymous changes	16	2.2599E-08	188	3.695E-07	16	1.8763E-08
Number of non-synonymous changes	49		251		82	
Number of total changes in chromosome I	35		389		55	
Number of synonymous changes in chromosome I	10	1.9743E-08	165	5.545E-07	8	1.7279E-08
Number of non-synonymous changes in chromosome I	25		224		47	
Number of total changes in chromosome II	30		50		43	
Number of synonymous changes in chromosome II	6	2.9776E-08	23	1.0889E-07	8	2.0525E-08
Number of non-synonymous changes in chromosome II	24		27		35	
Number of total changes in conjugative plasmid			1		0	
Number of synonymous changes in conjugative plasmid			0	0	0	0
Number of non-synonymous changes in conjugative plasmid			1		0	
Number of total changes in virulence plasmid					0	
Number of synonymous changes in virulence plasmid					0	0
Number of non-synonymous changes in virulence plasmid					0	

**Table 5:** Number of SNPs and MRs in pan genome (mutations per base pair per generation).

**Type**	**162**		**94385**		**CECT4604**	

	**Num**	**MR**	**Num**	**MR**	**Num**	**MR**
Number of total changes	205		534		299	
Number of synonymous changes	62		226		118	
Number of non-synonymous changes	143	3.97855E-08	308	1.32699E-07	181	8.68197E-08
Number of total changes in chromosome I	120		445		170	
Number of synonymous changes in chromosome I	38		187		65	
Number of non-synonymous changes in chromosome I	82	3.50537E-08	258	1.79713E-07	105	7.42757E-08
Number of total changes in chromosome II	85		89		129	
Number of synonymous changes in chromosome II	24		39		53	
Number of non-synonymous changes in chromosome II	61	5.06005E-08	50	8.0575E-08	76	1.09499E-07
Number of total changes in conjugative plasmid			1		0	
Number of synonymous changes in conjugative plasmid			0	0	0	0
Number of non-synonymous changes in conjugative plasmid			1		0	
Number of total changes in virulence plasmid					0	
Number of synonymous changes in virulence plasmid					0	0
Number of non-synonymous changes in virulence plasmid					0	

**Table 6:** Values of MR by genomic compartment.

**Genome compartment**		**MR (mutations per base pair per generation)**		

		**Min**	**Max**	**Average**
Core genome	Chro I+II	4.46876E-08	1.19951E-07	6.61887E-08
	Chr I	3.80919E-08	9.86927E-08	5.66973E-08
	Chr II	5.86515E-08	1.64957E-07	8.61048E-08
Accessory genome	Chro I+II	1.87626E-08	3.69504E-07	1.36955E-07
	Chr I	1.72788E-08	5.54496E-07	1.97173E-07
	Chr II	2.05253E-08	1.08890E-07	5.30636E-08
Pan genome	Chro I+II	3.97855E-08	1.32699E-07	7.77099E-08
	Chr I	3.50537E-08	1.79713E-07	7.82456E-08
	Chr II	5.06005E-08	1.12107E-07	6.93758E-08

**Table 7:** Confidence interval for MR at 95%.

**Compartment**	**Average**	**Lower limit**	**Upper limit**
Core genome	6.96636E-08	3.34584E-08	1.05869E-07
Accessory genome	1.29064E-07	0 (–3.72153E-08)	2.95343E-07
Pan genome	7.51104E-08	3.19755E-08	1.18245E-07
Chro I+II	9.36179E-08	0 (–1.48696E-09)	1.88723E-07
Chr I	1.10705E-07	0 (–3.91293E-08)	2.60540E-07
Chr II	6.95147E-08	3.12414E-08	1.07788E-07

### The ratio of non-synonymous to synonymous substitutions

Attending to the ratio of non-synonymous to synonymous substitutions (dN/dS), the type of selection forces on the evolution of this bacterial species can be evaluated (Kryazhimskiy and Plotkin, 2008). Values of dN/dS <1 indicate the purifying selection, a dN/dS = 1 indicates neutral evolution, and a dN/dS > 1 indicates positive selection. The values obtained for the studied strains are summarized in Table 8. Based on these results, strain 162 showed a very high ratio in all the genomic compartments and subdivisions of the genome, which is indicative of adaptive evolution. The same results could be found for strain 94385 but with a lower bias, but for strain CECT4604 not all the genomic subdivision showed this adaptative evolution as the core genome presented purifying (chromosome II and both chromosomes as a unit) or neutral selection (chromosome I). This strain, CECT4604, in the accessory genome showed the lowest ratio among the three studied strains.

**Table 8:** dN/dS ratio in the studied genomes.

**Compartment**	**162**			**94385**			**CECT4604**		

	**Core**	**Accessory**	**Pan genome**	**Core**	**Accessory**	**pan genome**	**Core**	**Accessory**	**Pan genome**
Chr_I-II	2.04347826	3.0625	2.30645161	1.5	1.33510638	1.36283186	0.97058824	5.125	1.53389831
Chr_I	2.03571429	2.5	2.15789474	1.54545455	1.35757576	1.37967914	1.01754386	5.875	1.61538462
Chr_II	2.05555556	4	2.54166667	1.4375	1.17391304	1.28205128	0.91111111	4.375	1.43396226

### Divergence time

The divergence time can be estimated using the MR and the number of changes in the genomes of the studied strains, specifically the core genome of the species. The core genome was described by Roig et al. (2018). The size of the core genome is presented in Table 2. The time was estimated using the following equation:


$$\begin{array}{l} Time\;\left(years\right)=\;\frac{\;Number\;of\;changes/Genome\text{\_}size}{num.\;generations\;by\;year\;x\;2\;x\;Mut\;rate}. \end{array}$$

The results for the studied strains in this study are shown in Table 9, and the divergence time for all strains studied in the core genome article (Roig et al., 2018) is shown in Supplementary Table 1.

**Table 9:** Time divergence.

		**ChroI**			**ChroII**			**ChroI-II**		

		**L2**	**L3**	**L4**	**L2**	**L3**	**L4**	**L2**	**L3**	**L4**
		**CECT4604**	**162**	**94385**	**CECT4604**	**162**	**94385**	**CECT4604**	**162**	**94385**
L2	CECT4604	0	668.268523	929.672309	0	436.012684	539.430109	0	569.064149	767.560967
L3	162	668.268523	0	873.924858	436.012684	0	524.133045	569.064149	0	727.655252
L4	94385	929.672309	873.924858	0	539.430109	524.133045	0	767.560967	727.655252	0

## Discussion

The MRs obtained for the core genome of both chromosomes of the three selected *V. vulnificus* isolates confirm the established theory on the evolution of both chromosomes in *Vibrio* (Cooper et al., 2010). According to this theory, chromosome II contains most of the genes involved in environmental adaptation, whereas chromosome I contains most of the housekeeping genes. Consequently, chromosome II is subject to a lower force of purifying selection and exhibits a higher evolutionary rate (dN and dS), a lower positive asymmetry of the rate distributions of orthologous sets, a lower codon usage bias, and, finally, a higher MR. For the accessory genome, only two of the strains studied, CECT4604 and 162, confirmed this theory; however, the third strain showed a higher MR for chromosome I than for chromosome II.

The first two strains belong to lineages II and III, while the third strain belongs to lineage IV, a minority lineage formed, so far, by European strains, so the result obtained cannot be standardized to this lineage until more strains are isolated and their genomes are sequenced. In any case, these results are consistent with the great biological and genetic diversity of this species (Oliver, 2015; Heng et al., 2017; Roig et al., 2018). For example, the *rtxA1* gene is present in this accessory genome, a gene encoding a multidomain toxin essential in virulence that is present on chromosome II (Roig et al., 2011, 2018). A lower chromosome II accessory genome MR could therefore pose adaptive value for those species that require genes in the accessory genome for survival.

Based on the dN/dS values, it appears that strains from L3 (162) and L4 (94385) are under positive selection pressure, the results for strain 162 showing the highest value while strain from L2 seems to be in “evolutionary neutrality”. This strain belongs to a clonal complex that emerged in fish farms in the 70s of the past century. This clonal complex appears to be quite stable, having been able to spread worldwide (Roig et al., 2018). Our results showed that the divergence time for chromosome I varied from 668.268523 to 929.672309 years, for chromosome II from 436.012684 to 539.430109 years, and for both chromosomes from 569.064149 to 767.560967 years. On the other hand, the divergence time for lineage II was 259 years, this value being the second lower number of years found for lineages with more than one representative. The most compact lineage in terms of divergence time is L3. This lineage emerged in the 90s of the last century. The divergence time between the strains studied is <1 year and the dN/dS rate is so high, indicating that this lineage is in full clonal expansion, which is consistent with previous studies.

These findings joined with the periodical outbreaks of *V. vulnificus* (López-Pérez et al., 2021) seem to indicate that the evolution of the species is not only influenced by the intrinsic MR but also be conditioned to external factors that exert selection pressure. This effect of selection pressure was previously studied by the authors in the selection of antibiotic-resistant clones under the presence of quinolones (Roig and Amaro, 2009).

Our results point to the fact that *V. vulnificus* is at a biological expansion level, with a lead to MR than other studied species; in *E. coli*, the calculated MR is 2.2 × 10^−10^ (Lee et al., 2012) mutations per base pair per generation, and in *Bacillus anthracis*, the calculated MR is 5.2 × 10^−10^ (Galloway-Peña et al., 2012). These findings alongside the colonization of new habitats due the climate change can lead to a rise in the number of infections, not only at the human level but at fish levels as well. This assumption is stated in the possibility of the emergence of new phenotypes with new putative host and virulence features.

Subsequent studies were conducted considering different culturing conditions, such as the presence of compounds such as blood or variations in temperature, which can induce differential expression of virulence-related genes and may potentially play a role in the MR, in this case by positive selection. This experiment could provide valuable insights into the evolution forces of the species. However, it is important to note that the objective of this study is focused solely on determining the MR under standard conditions. Therefore, future studies could explore the impact of various culturing conditions on both the MR and the differential expression of virulence-related genes. This could involve investigating a larger sample size or incorporating additional strains to enhance the generalizability and provide a more comprehensive understanding of the relationship between culturing conditions, MRs, and virulence-related gene expression.

## Data availability statement

The datasets presented in this study can be found in online repositories. The names of the repository/repositories and accession number(s) can be found below: https://www.ncbi.nlm.nih.gov/genbank/, PRJNA534103.

## Author contributions

FR designed the work and wrote the manuscript. FR, AA, and JC performed the experimental and statistical analysis. All the authors contributed to the discussion and improvement of the manuscript. All authors contributed to the article and approved the submitted version.
